# Pro-inflammatory markers as predictors of arterial thrombosis in aged patients with peripheral arterial disease post revascularization

**DOI:** 10.3389/fmed.2025.1615816

**Published:** 2025-07-24

**Authors:** Adriana A. Rodriguez Alvarez, Isabella F. Cieri, Mounika Naidu Boya, Shiv Patel, Aniket Agrawal, Sasha P. Suarez Ferreira, Christianah Alli, Shruti Sharma, Anahita Dua

**Affiliations:** ^1^Division of Vascular and Endovascular Surgery, Massachusetts General Hospital and Harvard Medical School, Boston, MA, United States; ^2^Department of Immunology, Tufts University School of Medicine, Boston, MA, United States

**Keywords:** inflammation, peripheral artery disease (PAD), tumor necrosis factor-alpha, interleukin-6, thrombosis

## Abstract

**Introduction:**

Inflammation occurs in the initial stage of arterial atherosclerosis and serves as the first step in thrombus generation, with elevated inflammatory markers predicting myocardial infarction in coronary artery disease patients. Inflammation is known to alter the course of multiple diseases and can thus, also impact recovery post-treatment and surgical outcomes. Yet, there is a paucity of data regarding the relationship between inflammatory biomarkers and arterial thrombotic potential in peripheral artery disease (PAD) patients post-revascularization. Our pilot study attempts to fill this gap by evaluating if the expression of inflammatory biomarkers in PAD patients correlates with the incidence of thrombotic events post-revascularization.

**Methods:**

Plasma samples were prospectively collected from PAD patients who underwent revascularization from 2021 to 2023 at monthly time points for 6 months from the procedure. Patients were followed for a total of 6 months post-procedure and those who experienced thrombotic events were identified. Nine patients with thrombotic events and 16 with non-thrombotic events along with 5 healthy volunteers were analyzed. Plasma samples were analyzed for the following pro-inflammatory markers: IL-1β (Interleukin-1 beta), IL-6, and TNF-*α* (Tumor Necrosis Factor - alpha), GM-CSF (Granulocyte-Macrophage Colony-Stimulating Factor), IFNγ (Interferon-gamma), IL-8, MCP-1 (Monocyte Chemoattractant Protein-1). The Kruskal-Wallis test was performed to compare bio-inflammatory marker levels between groups.

**Results:**

A total of 303 patients were enrolled, of which 59 had thrombotic events. There were no differences between medications or disease burden between groups. Levels of circulating IL-6 and TNF- *α* were significantly higher in the thrombosis cohort compared to the non-thrombosis cohort (55 vs. 38, *p* < 0.02) and (159 vs. 110, *p* < 0.02) respectively. Although there was a trend toward significance for IL-1β between the thrombotic cohort and non-thrombotic cohort, it did not reach statistical significance (18 vs. 11.5, *p* = NS). There was no difference observed in aspirin’s ability to dampen the inflammatory response between the two groups as all patients were on aspirin between the groups evaluated.

**Conclusion:**

Pro-inflammatory markers IL-6 and TNF-*α* are significantly increased in patients, 1 month prior to an arterial thrombotic event, as compared to patients without thrombotic events. These biomarkers could predict impending thrombosis in patients with PAD post-revascularization.

## Introduction

Peripheral arterial disease (PAD) is a critical condition stemming from atherosclerotic vascular disease, with its incidence continually rising, now affecting approximately 236 million individuals globally (1, 2). Individuals diagnosed with PAD suffer from a range of debilitating symptoms that span from diminished ambulatory capabilities to severe infarctions. Furthermore, PAD patients are at a significantly heightened risk of cardiovascular events, such as myocardial infarction and stroke, representing a profound public health issue (3–5).

Inflammation is integral to the initial stages of arterial atherosclerosis pathogenesis, initiating the cascade that leads to thrombus formation (6, 7). While extensive research has been conducted on the prognostic significance of elevated inflammatory markers in predicting myocardial infarction in patients with coronary artery disease (CAD) (8–12), the presence and implication of inflammation in PAD, particularly post-revascularization, remain underexplored (13, 14). Considering the distinct anatomy and increased systemic CRP levels suggesting a more pro-inflammatory profile of PAD relative to CAD, it is crucial to investigate the impact of inflammation on PAD-related outcomes independently (15).

The objective of this pilot study is to address this gap in knowledge by examining the differential expression of inflammatory biomarkers in PAD patients who develop thrombotic events post-revascularization compared to those who do not. This research is vital as it may uncover the inflammatory mechanisms that contribute to thrombus formation in PAD patients, which could lead to the development of specific therapeutic interventions to reduce such risks. Ultimately, this study aims to lay the groundwork for further extensive research by establishing a preliminary understanding of the relationship between inflammatory markers and thrombotic risk in the PAD population.

## Methods

### Study population

Patients ≥60 years with PAD, undergoing lower extremity revascularization procedures (endovascular, open, and/or combined) at a large single tertiary institution within the vascular surgery department were prospectively enrolled and followed clinically between January 2021 and December 2023. Exclusion criteria included the inability to provide informed consent, inability to undergo serial blood draws, pregnancy, prisoners, contraindication to antiplatelets, and bleeding disorders. In some cases, patients scheduled for revascularization did not undergo the planned intervention. If the index procedure did not result in successful revascularization, either due to a lack of a targetable lesion or an inability to endovascularly access a lesion, those patients were considered screen failures and excluded from the analysis. The study protocol, including the consent form, was reviewed and approved by the Institutional Review Board (IRB). All patients provided written consent before participation in the study, in accordance with ethical guidelines.

### Objective coagulation evaluation: quantitative analysis of inflammatory markers

A 5 cc blood sample was collected at 1-month, 3-month and 6-months post-procedure. Patients were followed for 6 months post-op to detect any thrombotic event. For patients who did not experience a thrombotic event, we used a sample collected at a comparable time point post-operative. As this was a pilot study, we randomly selected 9 patients with thrombotic events and 16 with non-thrombotic events from our cohort along with 5 healthy volunteers.

Plasma samples were analyzed for 23 inflammatory markers to ascertain inflammation in an unbiased and broad manner. The inflammatory markers analyzed in this study included GM-CSF(Granulocyte-Macrophage Colony-Stimulating Factor), IFN(Interferon) *γ*, IL (Interleukin)-1β, IL-1RA, IL-2, IL-4, IL-5, IL-6, IL-8, IL-10, IL-12p40, IL-12p70, IL-13, MCP-1(Monocyte Chemoattractant Protein-1), TNFα (Tumor Necrosis Factor – alpha), IFNα-2, IFNβ, IFNγR1, IFNε, IFNλ1, IFNλ2, IFNλ3, and IFNω. The samples analyzed were the last samples collected from the patient prior to a thrombotic event in that cohort. Plasma samples underwent a single freeze–thaw cycle. Blood was collected and immediately centrifuged to isolate plasma, which was then promptly frozen. Once an adequate number of samples had accumulated, all were thawed simultaneously and analyzed in a single batch.

Inflammatory biomarker concentrations were measured using multiplex bead-based immunoassays (Eve Technologies, Calgary, AB, Canada). The assays use polystyrene beads, each uniquely dyed and conjugated to a specific capture antibody, allowing simultaneous detection of multiple analytes in a single well. Samples were processed on a Bio-Plex 200 analyzer (Bio-Rad), which uses dual-laser flow cytometry to identify bead sets and quantify target concentrations via a streptavidin-phycoerythrin fluorescent reporter. Results were derived using standard curves for each analyte provided by the manufacturer (16).”

### Variables

Demographics, medications, procedure type and outcomes were documented between the thrombotic and non-thrombotic patients. Data analysis comparing quantitative level of inflammatory markers in patients who experience a thrombotic event within 6 months of a vascular intervention was compared with those who did not experience such an event within the same time period following the procedure. Thrombotic/stenotic events were defined as a composite of peripheral artery graft/stent thrombosis or stenosis, including radiographic evidence of graft/stent/native arterial intervention failure, reintervention to reestablish patent arterial flow, and major limb (above-knee or below-knee) amputation resulting from ischemia.

### Statistical analysis

Descriptive statistics were used to characterize the non-thrombosis and thrombosis groups. Mann–Whitney-*U* test was performed to evaluate differences in bio-inflammatory marker levels between groups to determine the potential for a risk-predictive relationship between these markers and the occurrence of a thrombotic event within 6 months post-revascularization.

## Results

### Study population and demographics

During the study period, 303 patients were enrolled of which 59 (19.4%) experienced a thrombotic event (“event”) while the rest did not (“non-event”). For the purpose of this research, 9 patients were selected at random from the event cohort and 16 patients were selected from the non-event cohort along with 5 healthy volunteers. The event cohort consisted of 77.8% males (7/9) and 22.2% females (2/9), while the non-event cohort comprised 56.3% males (9/16) and 43.8% females (7/16). (Table 1). The majority of patients in the entire study population were Caucasians with only 1 African American patient in the 25 studied (Table 1). Analysis of cardiovascular comorbidities showed comparable rates of hypertension (thrombotic: 88.9%, non-thrombotic: 81.3%, *p* = 0.26) and hyperlipidemia (thrombotic: 88.9%, non-thrombotic: 93.8%, *p* = 1.0) between groups. Coronary artery disease was observed in 88.9% of the thrombotic cohort compared to 50% of the non-thrombotic cohort, though this difference did not reach statistical significance (*p* = 0.4). There was no significant difference in the distribution of intervention types (endovascular, open, or combined) between the two groups (*p* = 0.86) Tobacco use did not exhibit any difference, with the majority of both cohorts being former smokers (Table 1).

**Table 1:** Demographics and comorbidities of study cohort (*N* = 25).

**Variables**		**Non-thrombosis *N* = 16**	**Thrombosis** ***N* = 9**	***p-value***
Sex				1
	Female			
	Study	7	2	
	Subjects	4	0	
	Controls			
	Male			
	Study	9	7	
	Subjects	1	0	
	Controls			
Race				0.36
	White			
	Study	16	8	
	Subjects	1	0	
	Controls			
	Black			
	Study	0	1	
	Subjects	1	0	
	Controls			
	Asian			
	Controls	1	0	
	Hispanic	1	0	
	Other			
	Controls	1	0	
Diabetes		3	6	0.03*
Hypertension		13	8	0.26*
Renal status				0.42*
	GFR Normal	8	4	
	GFR 60 to 89	5	2	
	GFR 30 to 59	3	2	
	GFR < 15 (on dialysis)	0	1	
Hyperlipidemia		15	8	1*
Coronary artery disease		8	8	0.4*
History of myocardial infarction		1	4	0.04*
History of DVT		4	2	1*
History of stroke		3	2	0.31*
Previous vascular intervention		6	6	0.23*
Tobacco use				0.69*
Never		3	1	
Past		10	8	
Current		3	0	
Type of intervention				0.86*
Endovascular		6	5	
Open		7	3	
Combined		3	1	

Data were presented as numbers (percentage). GFR = glomerular filtration rate; DVT = Deep vein thrombosis. Comparisons of variables between the thrombosis and non-thrombosis groups were performed using Fisher’s exact test. * Values indicate statistical significance.

### Vascular intervention type

In both cohorts, the majority of procedures were elective, for both Chronic Limb Threatening Ischemia (CLTI) and Intermittent Claudication. There was no significance in the type of intervention (Endovascular, Open, or Combined) (Table 1).

### Bio-inflammatory marker levels

**Figure 1:**
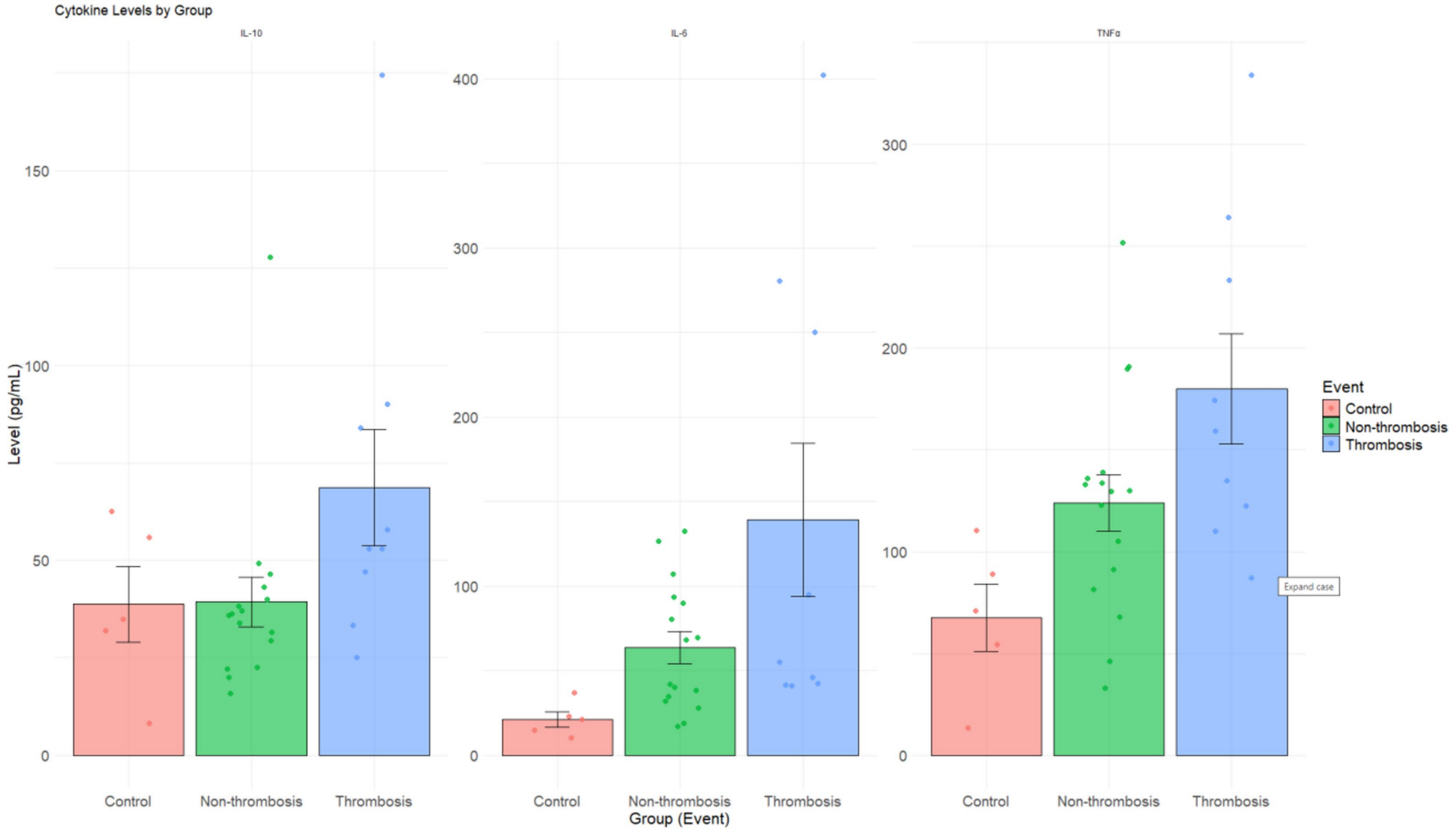
Cytokine levels by groups. Bar plots show mean ± standard error of cytokine levels (IL-10, IL-6, TNF*α*) in control (red), non-thrombosis (green), and thrombosis (blue) groups. Each dot represents an individual data point. All three cytokines demonstrated higher concentrations in the thrombosis group, indicating an amplified inflammatory response in thrombotic patients.

The thrombosis cohort exhibited significantly higher values of pro-inflammatory markers IL-6 [55 pg./mL vs. 38 pg./mL, *p* < 0.02] and TNF-*α* [159 pg./mL vs. 110 pg./mL, *p* < 0.02] compared to the non-event cohort, suggesting an increased risk of developing a thrombus in the former. While a trend trailing towards significance was observed for IL-1β, another pro-inflammatory marker, it failed to achieve statistical significance within the current cohort [18 vs. 11.5, *p* = 0.08] (Table 2). No statistically significant differences were observed in the remaining bio-inflammatory markers. When stratified by control, non-thrombosis, and thrombosis groups, IL-6 and TNF-*α* demonstrated a progressive increase across categories, consistent with a graded inflammatory response. IL-10 was also highest in the thrombosis group relative to non-thrombosis and controls, potentially indicating a compensatory anti-inflammatory effect (Figure 1).

**Table 2:** Inflammatory biomarkers.

**Inflammatory biomarkers (pg/mL)**	**Median**		**IQR**		***p*-value**
	**No thrombosis**	**Thrombosis**	**No thrombosis**	**Thrombosis**	
GM-CSF	46	46.5	33.5	9	0.625
IFNγ	14	18	9	13	0.504
IL-1β	11.5	18	6.5	8	0.086
IL-1RA	69	61.5	33.5	37	0.689
IL-2	17	17	10.5	14.5	0.824
IL-4	12	19	6.5	10	0.094
IL-5	24	32.5	18	22	0.056
IL-6	38	55	57.5	207.5	0.022*
IL-8	48.5	53	13	32	0.533
IL-10	36	53	13.5	37	0.019*
IL-12p40	15	17	11	4	0.326
IL-12p70	10	16	4	11	0.824
IL-13	12	15	7	10	0.125
MCP-1	1,083	1,374	880.5	1153.5	0.945
TNFα	110	159	62.5	110.5	0.028*
IFNα-2	9	9.5	2	1	0.193
IFNβ	12	12	11	5	0.79
IFNγR1	228.5	248	121	83	0.476
IFNε	14	12	5	7	0.209
IFNλ1	8	9	1	3	0.824
IFNλ2	14	10	7	6.5	0.063
IFNλ3	10.5	10.5	4.5	2	0.824
IFNω	9	8	12.5	3	0.722

Key: IL-6: Interleukin-6; TNF-α: Tumor Necrosis Factor-α; IL-1β: Interleukin-1 beta; GM-CSF: Granulocyte Monocyte-Colony Stimulating Factor; IFNγ: Interferon-γ. * Values indicate statistical significance.

## Discussion

Our pilot observational study analysis of 25 patients (9 with thrombotic events, 16 without) revealed distinct demographic parameters with the thrombotic group having a higher proportion of males (77.8% vs. 56.3%) with significantly higher rates of diabetes (*p* = 0.03) and history of myocardial infarction (*p* = 0.04). While both groups had comparable rates of hypertension (88.9% vs. 81.3%, *p* = 0.26) and hyperlipidemia (88.9% vs. 93.8%, *p* = 1.0), coronary artery disease was more prevalent in the thrombotic group (88.9% vs. 50%, *p* = 0.4). The type of intervention (endovascular, open, or combined) showed similar distribution between groups (*p* = 0.86), suggesting that surgical approach did not influence thrombotic outcomes.

Analysis of inflammatory cytokine profiles in our PAD patient cohorts revealed distinct patterns of immune activation. IL-1β demonstrated an upward trend (*p* = 0.086) but failed to achieve statistical significance in our pilot cohort. Notably, GM-CSF and IFNγ concentrations remained stable between cohorts, indicating selective rather than systemic pro-inflammatory cytokine upregulation in post-revascularization patients. This selective activation pattern suggests that specific molecular signatures may differentiate recovery versus relapse trajectories (17, 18).

Importantly, a point of relevance in this study is that all patients included in this analysis were maintained on aspirin therapy post-revascularization, and there were no significant differences in medication exposure or disease burden between the studied groups. This uniformity strengthens the interpretation of our cytokine profile data by minimizing pharmacologic confounding. Notably, prior work has shown that aspirin can influence cytokine dynamics, Brox et al. demonstrated that aspirin at physiologic concentrations enhanced TLR-stimulated production of IL-6 and IL-1β in human leukocytes through mechanisms independent of the COX-1 or COX-2 pathways traditionally attributed to aspirin use (19). Given this, the consistent aspirin exposure across all patients in our study ensures that observed differences in inflammatory marker levels, particularly IL-6 and TNF-*α*, are unlikely to be attributable to variability in anti-inflammatory medication use, but instead reflect true biologic divergence preceding thrombotic events.

The longitudinal Edinburgh Artery Study (*n* > 1,500, 12-year follow-up) established IL-6 as the predominant predictive inflammatory biomarker in PAD pathogenesis. As a primary immune regulator, IL-6 mediates hepatic acute phase protein production, particularly CRP. Multivariate analysis demonstrated that elevated IL-6 levels independently correlate with disease progression and diminished functional capacity. Clinical stratification revealed significantly higher IL-6 concentrations in patients with Severe Limb Ischemia compared to those presenting with claudication alone, establishing a quantitative relationship between cytokine levels and disease severity (20).

TNF-*α* demonstrated significant elevation in the event cohort. This monocyte-derived pro-inflammatory mediator plays a crucial role in atherosclerosis progression through multiple mechanistic pathways. Molecular analysis revealed increased monocyte TNF-*α* mRNA expression correlating with worsening claudication. Furthermore, patients exhibiting decreased Maximal Walking Time (MWT) showed concurrent elevation in both TNF-α gene expression and circulating protein levels, suggesting a direct molecular link between inflammation and functional impairment (21–23). The mechanistic relationship between IL-1β and atherosclerosis centers on its influence on plaque composition and stability. IL-1β, in conjunction with IFNγ, modulates Matrix Metalloproteinase expression, potentially destabilizing thrombotic plaques. Our findings suggest that the combinatorial assessment of IL-1β with IL-6/TNF-*α* may provide superior predictive value for re-thrombotic complications compared to IFNγ levels alone, which remained unchanged in our cohort. This observation aligns with genetic studies demonstrating increased IL-1β gene expression in coronary artery disease patients, supporting its role in atherosclerotic pathogenesis (18, 24).

## Limitations

One of our limitations was the small sample size due to budget constraints. Therefore, only univariate testing was able to be performed with limited power to detect differences. While we show a correlation between IL-6 and TNF- *α* (and possibly IL1 *β*) and post-surgical re-thrombotic events, outstanding gaps still exist in this new direction we have forged. For instance, it is unclear what the underlying source of these cytokines is in PAD patients with thrombotic complications. Furthermore, the impact of including an anti-inflammatory on the prospective outcomes is unknown though very attractive. The specific impact of IL-6/TNF- *α* on platelet biology that shapes potential thrombotic events even in the presence of anti-platelet therapy is an intriguing area of research. Moreover, it is also unclear when IL-6 and TNF- *α* levels become increased in PAD patients with revascularization, if it is directly proximal to the secondary thrombotic event or if they never see a decline in increased IL-6/TNF- α post-surgery. Thus, timing of these cytokines is critical in refining their predictive power. Finally, if these cytokines are sufficient to predict the thrombotic potential in revascularized PAD patients remains to be determined with an even broader assessment of immune mediators. If indeed, these cytokines are sufficient to predict the outcomes of revascularized patients, we will have inferred a roadmap to post-surgical success. We also acknowledge the lack of baseline samples at enrollment as a limitation. Baseline data would have strengthened comparisons and improved understanding of biomarker changes. Another limitation of our study is that we did not specifically analyze or adjust for the use of lipid-lowering therapy in relation to thrombotic outcomes, which may have influenced our findings. Future studies with a larger sample size should assess if increased IL-6, TNF and IL1b levels correlate and predict post-revascularization thrombotic events in the PAD population. Additionally, evaluate both the timing of cytokine elevation as a potential prognostic marker and the impact of lipid-lowering therapy on thrombotic risk in this population.

## Conclusion

Pro-inflammatory markers IL-6 and TNF-*α* are elevated in the months leading up to an arterial thrombotic event as compared to patients without thrombotic events. These biomarkers have a significant potential to be used as predictive markers of thrombosis in post-revascularization patients to monitor and determine their risk of re-thrombosis, thus might allow clinicians to intervene at the appropriate time to prevent the recurrence of such an event.

## Data Availability

The raw data will be made available upon reasonable request, pending approval from the corresponding author.

## References

[1] Fowkes FG Aboyans V Fowkes FJ McDermott MM Sampson UK Criqui MH. Peripheral artery disease: epidemiology and global perspectives. Nat Rev Cardiol. (2017) 14:156–70. doi: 10.1038/nrcardio.2016.179, PMID: 27853158

[2] About peripheral arterial disease (PAD) (2024). Centers for Disease Control and Prevention. Available online at: https://www.cdc.gov/heart-disease/about/peripheral-arterial-disease.html (Accessed June 28, 2024)

[3] Benjamin EJ Blaha MJ Chiuve SE Cushman M Das SR Deo R. Heart disease and stroke statistics-2017 update: a report from the American Heart Association. Circulation. (2017) 135:e146–603. doi: 10.1161/CIR.0000000000000485, PMID: 28122885 PMC5408160

[4] Golomb BA Dang TT Criqui MH. Peripheral arterial disease: morbidity and mortality implications. Circulation. (2006) 114:688–99. doi: 10.1161/CIRCULATIONAHA.105.593442 16908785

[5] Morrow K Dua A. Peripheral artery disease: where we are and where we are going. Semin Vasc Surg. (2022) 35:111–2. doi: 10.1053/j.semvascsurg.2022.04.001 35672100

[6] Stark K Massberg S. Interplay between inflammation and thrombosis in cardiovascular pathology. Nat Rev Cardiol. (2021) 18:666–82. doi: 10.1038/s41569-021-00552-1, PMID: 33958774 PMC8100938

[7] Aksu K Donmez A Keser G. Inflammation-induced thrombosis: mechanisms, disease associations and management. Curr Pharm Des. (2012) 18:1478–93. doi: 10.2174/138161212799504731, PMID: 22364132

[8] Madjid M Willerson JT. Inflammatory markers in coronary heart disease. Br Med Bull. (2011) 100:23–38. doi: 10.1093/bmb/ldr043, PMID: 22010105

[9] Lubrano V Balzan S. Consolidated and emerging inflammatory markers in coronary artery disease. World J Exp Med. (2015) 5:21–32. doi: 10.5493/wjem.v5.i1.21, PMID: 25699231 PMC4308529

[10] Blake GJ Ridker PM. Inflammatory bio-markers and cardiovascular risk prediction. J Intern Med. (2002) 252:283–94. doi: 10.1046/j.1365-2796.2002.01019.x, PMID: 12366601

[11] Pearson TA Mensah GA Alexander RW Anderson JL Cannon RO Criqui M. Markers of inflammation and cardiovascular disease: application to clinical and public health practice: a statement for healthcare professionals from the Centers for Disease Control and Prevention and the American Heart Association. Circulation. (2003) 107:499–511. doi: 10.1161/01.cir.0000052939.59093.45, PMID: 12551878

[12] Zakynthinos E Pappa N. Inflammatory biomarkers in coronary artery disease. J Cardiol. (2009) 53:317–33. doi: 10.1016/j.jjcc.2008.12.007, PMID: 19477372

[13] Paquissi FC. The role of inflammation in cardiovascular diseases: the predictive value of neutrophil-lymphocyte ratio as a marker in peripheral arterial disease. Ther Clin Risk Manag. (2016) 12:851–60. doi: 10.2147/TCRM.S107635, PMID: 27313459 PMC4892833

[14] Signorelli SS Anzaldi M Fiore V. Inflammation in peripheral arterial disease (PAD). Curr Pharm Des. (2012) 18:4350–7. doi: 10.2174/138161212802481273, PMID: 22390644

[15] Rein P Saely CH Silbernagel G Vonbank A Mathies R Drexel H. Systemic inflammation is higher in peripheral artery disease than in stable coronary artery disease. Atherosclerosis. (2015) 239:299–303. doi: 10.1016/j.atherosclerosis.2015.01.021, PMID: 25682027

[16] Eve technologies. Multiplexing LASER Bead Technology. https://www.evetechnologies.com/multiplex-and-msd-technologies/ (Accessed June 24, 2025)

[17] Subramaniyam V Waller EK Murrow JR Manatunga A Lonial S Kasirajan K. Bone marrow mobilization with granulocyte macrophage colony-stimulating factor improves endothelial dysfunction and exercise capacity in patients with peripheral arterial disease. Am Heart J. (2009) 158:53–60.e1. doi: 10.1016/j.ahj.2009.04.014, PMID: 19540392

[18] Enayati S Seifirad S Amiri P Abolhalaj M Mohammad-Amoli M. Interleukin-1 beta, interferon-gamma, and tumor necrosis factor-alpha gene expression in peripheral blood mononuclear cells of patients with coronary artery disease. ARYA Atheroscler. (2015) 11:267–74. 26715931 PMC4680074

[19] Brox R Hackstein H. Physiologically relevant aspirin concentrations trigger immunostimulatory cytokine production by human leukocytes. PLoS One. (2021) 16:e0254606. doi: 10.1371/journal.pone.0254606, PMID: 34428217 PMC8384208

[20] Tzoulaki I Murray GD Lee AJ Rumley A Lowe GD Fowkes FG. C-reactive protein, interleukin-6, and soluble adhesion molecules as predictors of progressive peripheral atherosclerosis in the general population: Edinburgh artery study. Circulation. (2005) 112:976–83. doi: 10.1161/CIRCULATIONAHA.104.513085, PMID: 16087797

[21] Poredoš P Šabovič M Božič Mijovski M Nikolajević J Antignani PL Paraskevas KI. Inflammatory and Prothrombotic biomarkers, DNA polymorphisms, MicroRNAs and personalized medicine for patients with peripheral arterial disease. Int J Mol Sci. (2022) 23:12054. doi: 10.3390/ijms231912054, PMID: 36233355 PMC9569699

[22] Gremmels H Teraa M de Jager SC Pasterkamp G de Borst GJ Verhaar MC. A pro-inflammatory biomarker-profile predicts amputation-free survival in patients with severe limb ischemia. Sci Rep. (2019) 9:10740. doi: 10.1038/s41598-019-47217-1 31341203 PMC6656730

[23] Ridker PM Cushman M Stampfer MJ Tracy RP Hennekens CH. Plasma concentration of C-reactive protein and risk of developing peripheral vascular disease. Circulation. (1998) 97:425–8. doi: 10.1161/01.cir.97.5.425, PMID: 9490235

[24] Trinh B Peletier M Simonsen C Plomgaard P Karstoft K Klarlund Pedersen B. Blocking endogenous IL-6 impairs mobilization of free fatty acids during rest and exercise in lean and obese men. Cell Rep Med. (2021) 2:100396. doi: 10.1016/j.xcrm.2021.100396, PMID: 34622233 PMC8484687

